# Transmission Distortion of MCT1 rs1049434 among Polish Elite Athletes

**DOI:** 10.3390/genes13050870

**Published:** 2022-05-12

**Authors:** Magdalena Dzitkowska-Zabielska, Aleksandra Bojarczuk, Małgorzata Borczyk, Marcin Piechota, Michał Korostyński, Jakub Grzegorz Adamczyk, Grzegorz Trybek, Myosotis Massidda, Paweł Cięszczyk

**Affiliations:** 1 Faculty of Physical Education, Gdansk University of Physical Education and Sport, 80-336 Gdansk, Poland; aleksandra.bojarczuk@awf.gda.pl (A.B.); pawel.cieszczyk@awf.gda.pl (P.C.); 2 Laboratory of Pharmacogenomics, Department of Molecular Pharmacology, Maj Institute of Pharmacology Polish Academy of Sciences, 31-343 Krakow, Poland; gosborcz@if-pan.krakow.pl (M.B.); marpiech@if-pan.krakow.pl (M.P.); michkor@if-pan.krakow.pl (M.K.); 3 Department of Theory of Sport, Józef Piłsudski University of Physical Education, 00-968 Warsaw, Poland; jakub.adamczyk@awf.edu.pl; 4 Department of Oral Surgery, Pomeranian Medical University, 70-111 Szczecin, Poland; g.trybek@gmail.com; 5 Department of Life and Environmental Sciences, University of Cagliari, 72-09124 Cagliari, Italy; myosotis.massidda@gmail.com

**Keywords:** polymorphisms, athletes, speed, endurance, HWE

## Abstract

Background: To date, nearly 300 genetic markers were linked to endurance and power/strength traits. The current study aimed to compare genotype distributions and allele frequencies of the common polymorphisms: *MCT1* rs1049434, *NRF2* rs12594956, *MYBPC3* rs1052373 and *HFE* rs1799945 in Polish elite athletes versus nonathletes. Methods: The study involved 101 male elite Polish athletes and 41 healthy individuals from the Polish population as a control group. SNP data were extracted from whole-genome sequencing (WGS) performed using the following parameters: paired reads of 150 bps, at least 90 Gb of data per sample with 300 M reads and 30× mean coverage. Results: All the analyzed polymorphisms conformed to Hardy–Weinberg equilibrium (HWE) in athletes and the control group, except the *MCT1* rs1049434, where allele T was over-represented in the elite trainers’ group. No significant between-group differences were found for analyzed polymorphisms. Conclusions: The *MCT1* rs1049434 transmission distortion might be characteristic of Polish athletes and the effect of strict inclusion criteria. This result and the lack of statistically significant changes in the frequency of other polymorphisms between the groups might result from the small group size.

## 1. Introduction

In the professional world of sport, victory is what counts. For many, competing at the Olympics is the pinnacle of an athlete’s career. There could be numerous athletes, but only a small group will achieve one of the most incredible milestones in sport. This means that, to be a top-level athlete, there has to be a factor/s contributing to their performance. Certainly, one of these is broadly defined genetics [1,2]. Factors found to be influencing athletes’ achievements have been explored in several studies. The heritability of athletic status (regardless of sport) was appraised at 66% [3]. The heritability of height, which is important for some sports, is estimated at 79% [4]. Body type reflecting general physique is also highly heritable [5], while these somatotypes are linked to the power or endurance athlete ability [6]. Nonetheless, there is enthralling evidence that a mutation in the erythropoietin receptor is a key factor in superior athletic performance in the case of Olympic glory [7]. However, central to the entire discipline of individual genes and variants of a particular DNA sequence (polymorphisms) and their genetic influence on athletic performance is the angiotensin-1 converting enzyme insertion/deletion (*ACE* I/D). The first is an insertion (I) or deletion (D) allele of 287 base pairs in intron 16 of the *ACE* gene [8] that has been reported to cause 47% of the variance in blood ACE levels [9]. Briefly, the I-allele seems to be associated with improved endurance performance, while the D-allele might offer advantages in strength/power-related sports [10]. Another performance factor is the *ACTN3* gene restricted to fast fiber. The X-allele has been considered advantageous in endurance, whereas the R in strength/power sports [11].

A large and growing body of literature has investigated the impact of many polymorphic variants on changes in athletic performance and conflicting results have been obtained [12,13]. The literature search uncovered approximately 300 genetic markers associated with athletic performance. These include 93 endurance- and 62 power/strength-related genetic markers. Most of the data were recognized with the use of the candidate gene approach [12] and were well-described in [11,14]. Much of the current literature pays particular attention to *MCT1*, *NRF2*, *MYBPC3* and *HFE* genes. *MCT1* gene (also known as SLC16A1) is a member of metabolic genes. Its expression is regulated by peroxisome proliferator-activated receptor-γ coactivator α (PGC-1α) in skeletal muscle [15]. *NRF2* encodes the nuclear respiratory factor 2 proposed to ameliorate pulmonary capacity, as well as ATP production while exercising [16,17]. *HFE* encodes a homeostatic iron regulator that regulates iron, and thus is linked to hemochromatosis [18]. Carriers of *HFE* polymorphisms have reduced peak oxygen uptake [19] and report tiredness or lack of energy [20]. *MYBPC3* codes for the thick-filament-associated protein cardiac myosin-binding protein C, and thus is linked with muscle sarcomeric structure and its contraction and relaxation [21]. *MCT1*, *NRF2* and *HFE* were listed in [12]. All four were also published elsewhere (*MCT1* in [22,23,24,25], *NRF2* in [26,27], *MYBPC3* in [28], and *HFE* in [18]). In addition, specific polymorphisms of those four genes contribute to athlete performance. That is: *MCT1* rs1049434 is linked to the athletic achievement of Russian, Japanese and Polish participants [29,30,31]; (2) *NRF2* rs12594956 is related to the athletic performance of Spanish subjects [26]; (3) there is an association between *MYBPC3* rs1052373 and the athletic ability of Europeans [28] and (4) *HFE* rs1799945 is related to the athletic performance of Russians and the Japanese [18]. Given that the aforementioned polymorphisms influence physical performance and are associated with athlete status, one might anticipate that they also would affect the Polish participants. Indeed, it is interesting whether or not some populations and geographical regions ensure success [32]. Therefore, the current study aimed to compare genotype distributions and allele frequencies of the selected polymorphisms in Polish elite athletes and nonathletic controls. The key research question of this study was whether or not the selected polymorphisms are important concerning elite athletes’ status. Our genetic approach was to characterize the underlying molecular mechanisms that determine athletic success.

## 2. Materials and Methods

### 2.1. Research Design

The study involved only highly elite Polish athletes—Olympic, World and European Championships medalists. The genotype data were compared to the results of analog investigations in the control group. The study was approved by Ethics Committee at The District Medical Chamber in Gdansk (KB-8/19). The research was conducted according to the Declaration of Helsinki. Written informed consent was obtained from all individual participants.

### 2.2. Participants

The study included 101 male elite Polish athletes (age at enrollment: 23.5 ± 5.9 years). Based on the previous methodology [22], athletes were divided into two groups according to metabolic requirements: the speed (*n* = 53) and the endurance (*n* = 48) athletes. The speed sports were sprints (100–400 m; *n* = 27), weightlifting (*n* = 24) and powerlifting (*n* = 2).

The endurance sports included 3000 m marathon (n = 7), cross-country skiing (*n* = 16), swimming (400–1500 m; *n* = 22), triathlon (*n* = 2) and rowing (*n* = 1). Inclusion criteria were based on the highest level of performance of the athletes. Scoring table (e.g., IAAF and FINA) classifications were used for the measurable sports (e.g., athletics, swimming or skating). The athletes with the best results that ranked them in the top 100 in a particular sports discipline in the world or Europe were included in the study group. Medal in the national championships or participation in the international competition at the European or World Championships were criteria for the non-measurable sports.

Controls were healthy individuals from the Polish population (*n* = 41, age at enrollment: 22.4 ± 6.3 years). These were taken from a cohort of families. To avoid confounding results due to relatedness, a kinship analysis based on the first two principal components of genotypes with MAF > 0.001 was performed (hail pc relate function). On a scale from 0 to 0.5, a kinship metric cut-off point value of 0.125 was assumed to remove related individuals. To participate in the study, the controls could not have had any medical history of any diseases affecting the heart or blood vessels and could not perform any competitive supervised sports training. All participants were unrelated and all were Caucasians of Polish origin. For DNA isolation, the buccal cells were collected using Copan FLOQSwabs (Copan Diagnostics, Inc., Murrieta, CA, USA).

### 2.3. Whole-Genome Sequencing (WGS) and Data Processing

WGS was performed externally by BGI Tech Solutions (BGI Co., Ltd., Hong Kong, China) using the following parameters: paired reads of 150 bps, at least 90 gb of data per sample with 300 M reads and 30× coverage. Fastq files were processed with Intelliseq Germline Pipeline 1.8.3 (https://gitlab.com/intelliseq/workflows, accessed on 9 December 2021) built with Cromwell (https://cromwell.readthedocs.io/en/stable/, accessed on 9 December 2021). Data processing was by fastQC assessment of the fastq file quality and by the alignment to Broad Institute Hg38 Human Reference Genome with GATK. Removal of duplicate reads was performed with Picard and, for recalibration of base quality, Phred scores GATK’s covariance recalibration was used.

Variants were called with GATK HaplotypeCaller and genomic variant calling files were obtained (gvcf). Files were then imported to Hail (0.2.62, https://hail.is/, accessed on 9 December 2021).

### 2.4. WGS Data Filtering and Annotation

All analyses described below were performed with Hail (0.2.62, https://hail.is/, accessed on 9 December 2021). Detailed information about file preparation/annotation and filtering is provided in the project Github repository (https://github.com/ippas/imdik-zekanowski-sportwgs, accessed on 9 December 2021). Briefly, all gvcf were combined into a sparse matrix table and genotyped.

Multiallelic variants were split and the Vcf file with all alternate allele calls in our cohort was filtered to exclude repeated and low-complexity sequences (UCSCRepeatMasker track). Further analysis involved only loci with more than 90% of GnomAD v3 samples with a DP of >1.

Variants were annotated with gnomAD (v3; https://gnomad.broadinstitute.org/, accessed on 9 December 2021) [30], CADD (CADD: Predicting the Deleteriousness of Variants throughout the Human Genome, Nucleic Acids Research, Oxford Academic, n.d.), Human Phenotype Ontology (HPO) [31], vep and a database of Polish genomes (https://naszegenomy.pl/, accessed on 9 December 2021). Preliminary principal component analysis of ~10 k genotypes (0.01%) revealed that one sample deviated more than 6 standard deviations (SDs) from the mean; thus, it was excluded. For additional quality control, the following filters were applied per each group: mean DP > 5, mean GQ > 50, max 3 samples with DP < 3 and max 3 samples with GQ < 30.

### 2.5. Statistical Analysis

Four genes were preselected for the analysis, with the focus on a selected SNP in each of the genes. For each of the tested variants, an HWE test was performed separately for the control and athlete groups. Fisher exact tests were performed between athletes and controls, endurance athletes and speed athletes and, additionally, between athletes and external database data: GnomAD non-Finnish Europeans and a database of Polish population allele frequencies (https://www.biorxiv.org/content/10.1101/2021.07.07.451425v1, accessed on 9 December 2021).

## 3. Results

Four genes and their polymorphisms with confirmed influence on speed or endurance were analyzed. A summary of SNPs for *MCT1, NRF2*, *MYBPC3* and *HFE* is provided in Table 1, including genetic variation, chromosomal position and gene location.

In the control group, *MCT1* rs1049434, *NRF2* rs12594956, *MYBPC3* rs1052373, and *HFE* rs1799945 conformed to Hardy–Weinberg equilibrium (HWE) *p* (*p*-value > 0.05). The following variants in the athlete group were also in equilibrium: *NRF2* rs12594956, *MYBPC3* rs1052373, and *HFE* rs1799945 (*p*-value > 0.05). However, the *MCT1* rs1049434 polymorphism was not (*p* = 0.029953). Table 2 compares the percentages of selected genotype frequencies, as well as experimental groups.

For analyzed polymorphisms, no significant between-group differences were found. However, rs1052373 of the *MYBPC3* was close to statistical significance between the endurance and speed groups. An additional 74 variants in the *MCT1* gene, 158 variants in the *NRF2 (GABPB1)*, 47 variants in the *MYBPC3* and 33 variants in the *HFE* passed all filters and had at least one nonreference call in our cohort. The frequency of alternate alleles and reference alleles was compared with Fisher’s exact tests for the following groups: speed vs. endurance athletes, all athletes vs. controls, all athletes vs. gnomAD non-Finnish Europeans and all athletes vs. Polish population (~900) samples. Differences between groups did not reach statistical significance after correction for multiple comparisons (Supplementary Table S1).

## 4. Discussion

This study aimed to investigate the frequency of *MCT1* rs1049434, *NRF2* rs12594956, *MYBPC3* rs1052373 and *HFE* rs1799945 gene variants among elite-level Polish athletes. The main finding of our study was that *MCT1* rs1049434 deviated from HWE. That might have been due to an excess of T allele only in the group of elite sportsmen. This deviation seems responsible for differences in genotypic distributions in the sports group for the rs1049434 marker. Disturbances of the HWE occur when natural selection favors a particular genotype giving a differential fitness. However, these cases are scarce in the literature, and large series are required to find statistically significant results. More often, Hardy–Weinberg disequilibrium (HWD) indicates population stratification [36,37,38]. The cause of HWE departure in our results is the genetic stratification, all the athlete individuals included in this study were strictly selected due to their master sports achievements. In the control group, the HWE was preserved. This indicates that the distribution of the alleles is constant when mating is random, with no disruptive circumstances and no strict selection criteria due to the trait/phenotype [38].

Some researchers report significant associations with the status of being an elite endurance athlete, e.g., *NRF2* AG genotype and the G allele. The study involved 119 male and 36 female Israeli participants [39]. Similarly, an association between *MYBPC3* polymorphism and endurance achievements was found in a large European cohort of 645 men and 151 women [28]. Likewise, the study of 83 superior male French road cyclists revealed a mutation in the *HFE* gene [40] and a mutated *MYBPC3* in Japanese athletes with abnormal ECG [41]. It is acknowledged that mutations and polymorphisms are not interchangeable, but there are cases of a rare disease allele in some populations becoming a polymorphism in other ones.

In contrast, a less consistent result was shown for *ACE* I/D SNP in 99 male and 22 female Israeli individuals performing endurance sports [42]. The same conclusion applies to *ACE* I/D in the study including 51 Italian athletes [43]. Furthermore, *PPARD* T294C/*PPARGC1A* Gly482Ser SNP was related to endurance only in one of a few performances of 111 Japanese athletes [44], highlighting a sample size issue. The literature often describes that it might not be possible to investigate the significant relationships because the sample size is too small [23,26,45,46].

There were no significant differences in the frequency of the analyzed genotypes between the speed and endurance groups. The MCT1 rs1049434 TT genotype was more frequent in athletes, but this was not statistically different from the control group. This was most likely due to the small sample size. However, it should be noted that the T allele in the study is not a minor allele. The T allele appears more frequent in our study. This is in agreement with the National Center for Biotechnology Information (NCBI) data, indicating the prevalence of the alternate T allele in the European population (https://www.ncbi.nlm.nih.gov/snp/rs1049434, accessed on 4 March 2022 ).

Our WGS analysis that the frequency of TT genotype seems to be increased in the sportsmen group corroborates earlier findings of Guilherme et al. reporting that the T allele was major, whereas the A allele was mutated. This was related to the *MCT1* gene Glu490Asp SNP and endurance participants [22]. Our findings are thought to be opposite to those of Sawczuk et al. [31]. The aforementioned authors used a method of Fedotovskaya [29], who used PCR-RFLP and of whom our alleles assignment is inversed, and the A allele in this study is the same as the T allele in [29,31]. In [31], the T allele was the minor allele, although the odds of having the *MCT1* TT genotype were almost threefold higher for top-level sprint/power athletes. However, according to the genotypic frequencies, our data are believed to be in line with the study of Fedotovskaya et al. [29]. The authors of [29] observed an association of AA genotype with endurance athlete status. Based on genotypic frequencies, these data corroborate our observation regarding TT genotype in sportsmen vs. controls.

The *MCT1* (*SLC16A1*) gene is located at 1p13.2 and encodes monocarboxylate (lactate/pyruvate) transporter [47]. *MCT1* is involved in the transfer of lactate and protons in the skeletal muscles, thereby affecting muscle contraction [25,47]. A common A1470T (Glu490Asp) polymorphism (rs1049434) results in the substitution of glutamic acid for aspartic acid and regulates lactate rates. During intense circuit weight exercises, the lactate transport is diminished in the T allele carriers at 60–65% [48] and blood lactate build up [49]. Several subsequent studies assessing muscle injury and body composition also demonstrated over-representation of the T allele and TT genotype of the *MCT1* rs1049434 polymorphism among athletes [50,51]. However, in the case of [50,51], due to the techniques used in those studies, the key point needs to be underlined. That is, the present study could not directly compare *MCT1* TT results because of the alleles’ designation and, thus, their inversion. This implies that it would be the TT genotype in [50,51] that should be compared with our AA and vice versa. This also points to the conclusion that our data are not in agreement with the aforementioned studies. This study has also been unable to demonstrate the accordance with Saito et al. that showed an over-representation of the T allele among Polish climbers [24].

Our genotypes and allele frequency analyses of the *NRF-2* rs12594956 and *HFE* rs1799945 did not confirm the published data, as no statistical significance was observed.

Concerning the *NRF-2* rs12594956, Eynon et al. estimated the likelihood of having the AA genotype was higher in elite endurance athletes as compared with controls and elite power athletes [26,52]. The influence of rs12594956 on the regulation of *NRF2* gene and/or protein expression needs to be elucidated since this particular SNP is located in an intron region and might contribute to aerobic fitness phenotype by improving respiratory capacity and increasing the rate of ATP production during exercise [16,27]. In terms of *HFE* H63D (rs1799945), this polymorphism is strongly associated with elite endurance athlete status, regardless of ethnicity and aerobic capacity. The transferrin receptor encoded by the *HFE* gene plays a primary mode in iron storage through regulation of the hepcidin hormone, leading to increased hemoglobin levels [46]. The meta-analysis using cohorts of French, Japanese, Spanish, and Russian athletes showed a significant prevalence of the CG/GG genotypes in endurance athletes compared to controls. Individuals with the G allele (homo- and heterozygotes) show higher circulating iron concentrations, improved endurance performance and increased VO_2_peak [18,20].

There are a few limitations to this study. Due to the relatively modest sample size (n = 142), it is likely that statistical power was not sufficient to detect associations. As it was not possible to expand the group of elite athletes in the respective nationality, it would be essential to have a larger control group in the future. A nonrandom selection of study groups might result in selection bias. These limitations are hard to overcome as our study enrolled only athletes with the highest level of performance and of Polish origin, which is a quite small and homogenous group.

## 5. Conclusions

Our study demonstrated that the T allele of the *MCT1* rs1049434 appeared over-represented in the athletes’ group. The over-representation is only a suggestion since there was no difference in Fischer’s exact test between the groups.

## Figures and Tables

**Table 1:** The characteristics of single-nucleotide polymorphisms for *MCT1*, *NRF2*, *MYBPC3* and *HFE* genes [22,33,34].

	*MCT1* rs1049434	*NRF2* rs12594956	*MYBPC3* rs1052373	*HFE* rs1799945
Locus	chr1:112913924	chr15:50329637	chr11:47333236	chr6:26090951
Alleles	A > T	C > A	C > T	C > G
CADD	1.501	0.723	4.92	15.61
Allele frequency in the Polish population (obtained from https://www.biorxiv.org/content/10.1101/2021.07.07.451425v1, accessed on 9 December 2021)	0.597	0.512	0.311	0.152
Gene Location	exon	intron	exon	exon
Functional Consequence	missense variant	-	synonymous variant	missense variant

CADD—a well-described genome-wide variant prioritization tool [35].

**Table 2:** Genotype distribution of the *MCT1* rs1049434, *NRF2* rs12594956, *MYBPC3* rs1052373, and *HFE* rs1799945 polymorphisms and comparisons between experimental groups.

					*MCT1* rs1049434					
Genotype Frequency (%)					Fisher, *p*-value				HWE, *p*-value	
Group	n	AA	AT	TT	Sp vs. C	E vs. C	S vs. C	E vs. S	Sp	C
Sp	101	20.8	37.6	41.6	0.3525	0.1252	0.8826	0.1535	0.0300 *	0.1961
E	48	16.8	35.4	47.8						
S	53	24.5	39.6	35.9						
C	41	24.4	43.9	31.7						
					*NRF2* rs12594956					
					Fisher, *p*-value				HWE, *p*-value	
Group	n	CC	CA	AA	Sp vs. C	E vs. C	S vs. C	E vs. S	Sp	C
Sp	101	17.8	57.4	24.8	0.7918	0.1252	0.8826	0.1529	0.1393	0.6038
E	48	18.8	56.3	25.0						
S	53	17.0	58.5	24.5						
C	40	20.0	57.5	22.5						
					*MYBPC3* rs1052373					
					Fisher, *p*-value				HWE, *p*-value	
Group	n	CC	CT	TT	Sp vs. C	E vs. C	S vs. C	E vs. S	Sp	C
Sp	101	43.6	42.6	13.9	0.7831	0.6434	0.2706	0.0770	0.4492	0.3881
E	48	37.5	41.7	20.8						
S	53	49.1	43.4	7.5						
C	40	40.0	45.0	15.0						
					*HFE* rs1799945					
					Fisher, *p*-value				HWE, *p*-value	
Group	n	CC	CG	GG	Sp vs. C	E vs. C	S vs. C	E vs. S	Sp	C
Sp	101	74.3	23.8	2.0	0.1556	0.1018	0.4480	0.4169	0.8432	0.5380
E	48	79.2	18.8	2.1						
S	53	69.8	28.3	1.9						
C	41	68.3	22.0	9.8						

Sp—sport, E—endurance, S—speed, C–control, * statistical significant.

## Data Availability

The data presented in this study are available upon request from the corresponding author. The data are not publicly available due to ethical reasons.

## Supplementary Materials

The following supporting information can be downloaded at: www.mdpi.com/article/10.3390/genes13050870/s1, Table S1: Supplementary Table S1.
